# Mesenchymal Stem Cells and Reticulated Platelets: New Horizons in Multiple Myeloma

**DOI:** 10.3390/hematolrep16040070

**Published:** 2024-11-23

**Authors:** Cristian Alejandro Mera Azaín, Johan Leandro Vargas Pasquel, Sandra Milena Quijano Gómez, Viviana Marcela Rodríguez-Pardo

**Affiliations:** Grupo Inmunobiología y Biología Celular, Facultad de Ciencias, Pontificia Universidad Javeriana, Bogotá 110111, Colombia; mera.cristian@javeriana.edu.co (C.A.M.A.); johanvargas@javeriana.edu.co (J.L.V.P.); squijano@javeriana.edu.co (S.M.Q.G.)

**Keywords:** multiple myeloma, mesenchymal stem cells, reticulated platelets

## Abstract

Multiple myeloma (MM) is a malignant plasma cell disorder characterized by the accumulation of abnormal plasma cells in the bone marrow. Mesenchymal stem cells (MSCs) and reticulated platelets (RPs) have been implicated in the pathogenesis of MM. This narrative review aims to explore the role of MSCs and RPs in the pathophysiology of MM, particularly their clinical use as possible variables of prognostic value in this hematologic neoplasia. The interaction between MSCs and MM cells within the bone marrow microenvironment supports MM cell survival, proliferation, and drug resistance. MSCs contribute to the development and maintenance of MM through the secretion of various factors, including cytokines, chemokines, and growth factors. Moreover, RPs, young and highly reactive platelets, have been implicated in promoting angiogenesis, tumor growth, and metastasis in MM. Several studies show that cells such as MSCs and platelets participate actively in the biology of the disease. Still, in clinical practice, they are not considered part of evaluating affected patients. In this review, we explore the possibility of including the evaluation of MSCs and PRs in the clinical practice for patients with MM as part of the strategies to improve the outcomes of this disease.

## 1. Introduction

Multiple myeloma (MM) is a hematological malignancy of clonal bone marrow plasma cells, characterized by uncontrolled growth and overproduction of nonfunctional immunoglobulins (Igs). It belongs to the plasma cell disorders group, including MGUS, smoldering myeloma, solitary bone plasmacytomas, and amyloidosis [1,2]. The etiology involves exposure to chemicals, toxins, radiation, and genetic alterations, such as mutations in growth-related genes, chromosomal translocations, and hyperdiploidy [3,4]. MM is the second most common hematologic cancer after non-Hodgkin’s lymphoma, mainly affecting those over 65. In 2020, MM had a global incidence and mortality of 1.8 and 1.1 per 100,000, with higher rates in men [5]. Patients often exhibit “CRAB criteria” (hypercalcemia, renal failure, anemia, bone disease) and symptoms like fatigue, bone pain, infections, neurological issues, and displacement of bone marrow cells by malignant plasma cells [3,6,7]. A defining feature is osteolytic bone disease (OBD), caused by altered bone homeostasis with increased osteoclast activity and suppressed osteoblasts, leading to bone resorption and lesions, primarily in vertebrae and long bones. At diagnosis, 85% of patients show bone involvement, correlating with the tumor burden and prognosis [8]. Another hallmark is hyperviscosity syndrome from excessive Ig production, causing nephron blockage, blood flow impairment, and tissue hypoxia. Clinically, it manifests as neurological, ophthalmological, and cardiovascular issues, including heart failure [9].

The International Myeloma Working Group defines MM diagnosis by ≥10% clonal bone marrow plasma cells or extramedullary plasmacytoma, alongside genetic findings like trisomy 21, del(17p), and translocations involving chromosome 14 (14q32) [2,10,11,12] (Table 1). Secondary genetic changes, such as del17p13, RAS mutations, and MYC translocations, impact the staging and prognosis [11,12,13,14,15]. Chromosome 13 abnormalities, present in 50% of cases, manifest as monosomy (85%) or deletions (15%) and are linked to high-risk features like t(4;14) rather than being independent prognostic factors. Its role is tied to non-hyperdiploid MM and tumor expansion [13,14] (Table 2).

## 2. Role of MSCs in the Development, Maintenance, and Progression of Multiple Myeloma

**Figure 1:**
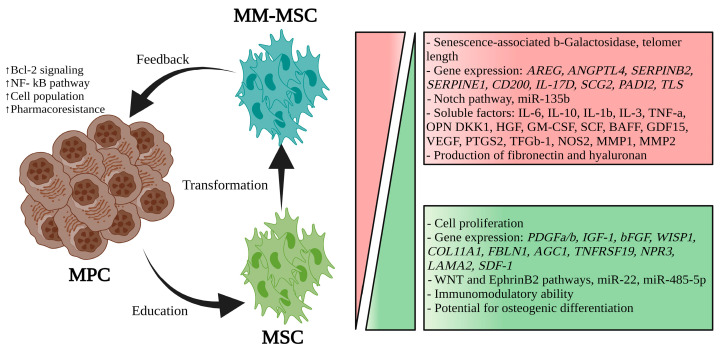
Comparison of MSCs and MM-MSCs (Adapted from Xu et al. (2018) [23] and Garcia-Gomez et al. (2014) [24]). The MPCs “educate” the MSCs to transform them into MM-MSCs by modifying their genotype and phenotype. In turn, the MM-MSCs promote the survival of MPCs through direct contact and soluble factors [23,24]. (↑: increase) (red box: increase, green box: decrease) (MPC: myeloma plasma cells, MSC: mesenchymal stem cells, MM-MSC: Mesenchymal stem cells associated with multiple myeloma).

MSCs are non-hematopoietic multipotent cells derived from the mesoderm and ectoderm, capable of differentiating into mesodermal (osteocytes, adipocytes, chondrocytes), ectodermal (neurocytes), and endodermal (hepatocytes) lineages [14]. First isolated from the BM, MSCs are also found in adipose tissue, endometrium, amniotic fluid, and umbilical cord, supporting hematopoiesis, immunity, and tissue regeneration [16,17]. In vitro, MSCs express CD73, CD90, and CD105 but lack CD14, CD34, CD45, and HLA-DR; they differentiate into chondrocytes, osteocytes, and adipocytes [18]. ISO Standard 24651 revised their phenotypic definition [18,19]. MSCs modulate immunity through TLR signaling: TLR4 activation induces a pro-inflammatory phenotype (MSC1), while TLR3 promotes an immunosuppressive phenotype (MSC2) [20]. MSCs migrate to inflammation, damage, or tumor sites through adhesion molecules (e.g., VLA-4) and growth factors (e.g., SDF-1) [19,21,22]. In MM, MSCs (MM-MSCs) acquire characteristics that enhance tumor growth, survival, and resistance [11,23]. Educated by MM cells, MM-MSCs remodel the BM microenvironment via BMP/TGF-β signaling, promoting immune evasion and drug resistance (Figure 1) [24]. Corre et al. identified 145 differentially expressed genes in MM-MSCs, linked to angiogenesis, tumor growth, and impaired osteogenesis. MM-MSCs secrete IL-6, GDF15, and adhesion molecules like ICAM-1 and VCAM-1, activating the NF-κB pathway to enhance proliferation, metastasis, and pharmacoresistance (Figure 1) [25,26]. MM-MSCs also produce cytokines (e.g., IL-1β, VEGF) that support tumor survival and pathological BM lesions [11].

MM-MSCs secrete exosomes with miR-146a, activating pathways like NF-κB and MAPK and enhancing tumor survival and migration (Figure 1) [27,28]. They exhibit an inflammatory phenotype (iMSCs), producing cytokines like IL-6 and CXCL8 and contributing to relapse and therapy resistance [29]. MM-MSCs also boost MM cell “stemness” by activating BTK signaling [30,31] and transfer mitochondria via tunneling nanotubes (TNTs), enhancing oxidative phosphorylation and chemoresistance [32,33]. BM-MSCs secrete soluble factors like IL-6 and IL-8, promoting proteasome inhibitor resistance via NF-κB activation [34]. CXCL12/CXCR4 signaling facilitates mitochondrial trafficking, enhancing MM cell survival [35]. CAR T-cell therapies targeting BCMA show promise but face challenges from MSC-driven immune evasion. Strategies like dual-target CARs, PD-1 inhibitors, and armored CAR T-cells aim to counter MSC-mediated resistance and improve outcomes [36,37].

## 3. MSCs and Their Role in the Development of Osteolytic Bone Disease and Distant Migration of Malignant Plasma Cells in Multiple Myeloma

**Figure 2:**
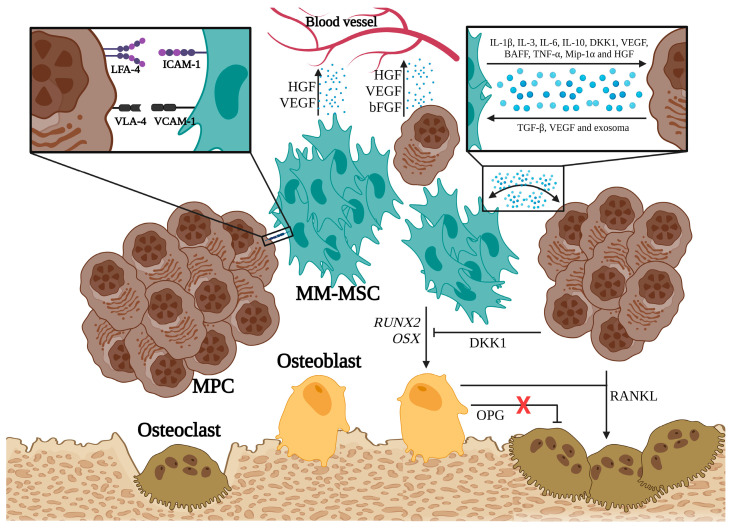
MM-MSCs implicated in the development of bone marrow disease (Adapted from Garcia-Gomez et al. (2014) [24]). (**X**: removal of inhibition) (RUNX2: Runt-related transcription factor 2, OSX: Osterix, DKK1: Dickkopf-related protein 1, OPG: Osteopontin, RANKL: Receptor activator of nuclear factor kappa-Β ligand) (activation: →, inhibition: 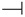).

Several mechanisms have been described as linking MM-MSCs to the development of osteolytic bone disease in patients with MM. Several studies indicate that the Notch signaling pathway is very active in MM-MSCs, where it downregulates the expression of RUNX2, a critical factor for their osteogenic differentiation [23,38]. In addition, MM-MSCs exhibit an abnormal expression of some molecules, such as miR-135b, which correlates inversely with bone alkaline phosphatase activity [24]. PPARγ2 mediates another mechanism associated with the development of osteolytic lesions; this transcription factor is involved in the regulation of fatty acid storage, glucose metabolism, and activation of adipogenesis and is essential in the suppression of osteoblastogenesis, which is highly active in MM-MSCs [39]. Finally, HGF secreted by MM-MSCs and osteoclasts is another relevant factor in the progression of the osteolytic bone disease. HGF interacts with its receptor on both osteoblasts and osteoclasts. In osteoblasts, HGF prevents the transcription of RUNX-2 and OSX, disrupting their differentiation; however, it also induces the secretion of IL-11, which promotes osteoclast activation (Figure 2).

As MPCs migrate distantly, they remodel their new microenvironment and convert it into a space that is favorable for their proliferation and survival. It has been proposed that MM-MSCs actively participate in the distant migration of MPCs by regulating GAP junctions, which are made up of molecules such as Cx43 [40]. The MM-MSCs express a higher level of Cx43 than normal MSCs; this feature enhances the migration of MPCs in vitro. Another mechanism involved in the distant migration of neoplastic cells is the secretion of matrix metalloproteinase-9 (MMP-9) by MM-MSCs, which facilitates the mobilization of MPCs into the extracellular matrix [41]. Rethnam, M, et al. demonstrated that normal BM-MSCs secrete elevated levels of TIMP-1 (MMP-9 inhibitor); however, in the presence of MPCs, the TIMP-1 levels decrease, and the production of MMP-9 by MSCs is favored [42].

## 4. Biological Features of RPs and Their Role in the Bone Marrow Microenvironment in MM

Reticulated platelets (RPs), also known as immature platelets, are recently released from bone marrow megakaryocytes and possess elevated mRNA content, making them the youngest, most reactive, and largest platelets. They have a lifespan of 24–36 h, during which RNA degrades and their mean platelet volume decreases. Their reactivity and protein synthesis capacity make them key markers of megakaryopoiesis and platelet turnover. Automated hematology analyzers have standardized RP quantification, enhancing their clinical application. Elevated RPs are linked to conditions like acute coronary syndrome, stroke, and sepsis, serving as diagnostic and prognostic biomarkers [43,44]. RPs exhibit higher thrombotic activity than mature platelets, with increased ADP, ATP, serotonin release, and expression of glycoproteins (GPIb, GPIIb/IIIa) and adhesion molecules (P-selectin), improving environmental adhesion [45]. Lador, A, et al. noted higher activation marker expression (P-selectin, annexin-V) and protein translation in RPs [46].

Platelet–tumor interactions, including platelet–tumor cell aggregates, are mediated by integrins, selectins (P-selectin), and Ig superfamily proteins. In MM, heterotypic adhesion via P-selectin and PECAM-1 promotes tumor survival and metastasis by releasing mediators like ATP and MMPs, degrading the extracellular matrix and enabling invasion [47,48]. Schumacher, D, et al. highlighted that ATP released by activated platelets eases tumor migration, while CD97 triggers granule secretion, thus enhancing invasiveness [49,50].

MM patients exhibit reduced platelet counts and function, with prolonged thrombin lag times despite hypercoagulability. Platelet dysfunction contributes to the paradoxical coagulopathy in MM, characterized by elevated bleeding and thrombosis risks [51]. O’Sullivan et al. demonstrated hyperactivation of platelets in MGUS and MM, linking this to increased thrombotic risk, although platelet exhaustion reduces responsiveness in MGUS [52]. In contrast, RPs remain reactive, interacting with MPCs via molecules like ADP and P-selectin, promoting tumor survival and migration [49]. RPs’ high RNA content enhances reactivity, aggregation, and granule secretion, regulating the gene expression in other cells through extracellular vesicles. MM treatments, such as IMiDs, increase platelet reactivity, exacerbating the thrombotic risk [53,54]. CAR-T therapy also alters platelet function, affecting lipid metabolism and platelet-activating factor synthesis, contributing to thrombotic complications [55,56].

RPs play a central role in MM progression, promoting angiogenesis, tumor growth, and metastasis while increasing the thrombotic risk. Monitoring RPs offers prognostic value and aids in tailoring therapies to manage bleeding and thrombosis risks in MM patients [57].

## 5. MSCs and RPs as Valuable Prognostic Variables in MM

MSCs have been observed to interact with cancer cells at various stages of cancer progression, influencing their invasive behavior and promoting metastasis formation. In this regard, four main mechanisms have been described [58,59,60]: (i) Induction of a pro-metastatic state—MSCs have the ability to modify the tumor microenvironment and direct tumor cells towards an invasive pro-metastatic state. It has been observed that MSCs promote the migration, invasion and extravasation of tumor cells, thus facilitating their spread to other organs, possibly through the promotion of an epithelial–mesenchymal transition (EMT) or through the induction of phenotypic changes in tumor cells that are associated with modifications in the profile of adhesion molecules, among others. (ii) Interaction with the tumor stroma—MSCs can migrate toward the stroma of primary tumors and also toward metastatic sites, promoting the generation of cancer-associated fibroblasts (CAFs), which have been shown to actively participate in metastatic processes. (iii) Influence on the formation of metastatic niches—It has been suggested that MSCs may participate in the creation and maintenance of metastatic niches, which are favorable microenvironments for the survival and growth of metastatic tumor cells. These niches provide conditions that are conducive to the colonization of cancer cells at distant sites. Kaplan et al. have shown that bone marrow stromal cells expressing VEGFR1+ migrants form pre-metastatic complexes before the arrival of tumor cells. (iv) Interaction with cells of the immune system—MSCs have the ability to modulate the immune response, which may influence the ability of the immune system to detect and eliminate metastatic tumor cells. In addition, MSCs can suppress the antitumor immune response, facilitating the immune evasion of cancer cells and their ability to form metastases.

Although the association between MSCs and multiple myeloma has been demonstrated [24], the evaluation of MSCs as a possible variable of prognostic value for metastasis is not used in clinical practice. Interestingly, Van der Velden et al. have shown that in different types of cancer such as sarcoma and colon and prostate cancer, the number of endogenous mesenchymal stem cells in the peripheral blood is increased, which demonstrates that the evaluation of these cells in cancer could have an important clinical impact [61].

Particularly in MM, it would be very useful to evaluate the number of MSCs in the peripheral blood and/or bone marrow of newly diagnosed patients and after chemotherapy due to the association of these cells with metastasis in other cancer models, where MSCs migrate to a location away from the tumor cell in order to generate pre-metastatic niches [62,63]. Additionally, it would be very useful to define the transcriptomic and/or proteomic profile of MSCs in each patient with MM as an approach to personalized medicine due to MM-MSC dysfunction and its association with the appearance of lytic lesions and disease progression.

Regarding the role of platelets as a variable of prognostic value in MM, in clinical practice, only their quantification is considered useful [64,65]; however, other aspects such as the determination of reticulated platelets or their activation are not currently evaluated. Previous studies have shown that platelets may participate in the pathophysiology of MM through four mechanisms [46,49,50,52]: (i) Markers of Active Thrombopoiesis—Reticulated platelets are younger and more active than mature platelets, reflecting accelerated thrombopoiesis. In MM, where hematopoiesis is often disrupted, elevated levels of reticulated platelets may indicate active platelet production in response to thrombocytopenia or platelet destruction caused by the disease. (ii) Role in the Tumor Microenvironment—Reticulated platelets have been implicated in promoting angiogenesis and tumor growth, contributing to the microenvironment that supports MM progression. These platelets may help facilitate tumor cell extravasation and modify the bone microenvironment, which is significantly affected in MM. (iii) Indicator of Platelet Activation—Since reticulated platelets are more reactive, elevated levels of these cells may correlate with heightened platelet activation, which has been observed in MM patients. Platelet activation is linked to a higher risk of thrombotic complications, adding to the disease burden in MM. (iv) Prognostic Implications—Increased levels of reticulated platelets in MM may reflect more active disease and a worse prognosis. Monitoring reticulated platelet counts could provide a useful tool for tracking disease progression and treatment response in clinical practice.

## 6. Conclusions

In conclusion, both mesenchymal stem cells (MSCs) and reticulated platelets (RPs) play crucial roles in the development, maintenance, and progression of multiple myeloma (MM). MSCs create a supportive microenvironment for myeloma cells by secreting cytokines, chemokines, and growth factors that promote cell survival, proliferation, and drug resistance. Moreover, the genetic abnormalities and phenotypic characteristics of MSCs in MM patients further contribute to the aggressiveness of the disease. RPs, as young, highly reactive platelets, are implicated in enhancing angiogenesis, tumor growth, and metastasis.

Looking towards the future, further research into the integration of mesenchymal stem cells (MSCs) and reticulated platelets (RPs) in multiple myeloma (MM) holds considerable potential for clinical advancement. Ongoing clinical trials are exploring the therapeutic potential of MSCs, particularly their capacity to modulate the tumor microenvironment and influence disease progression through tumor-targeted drug delivery, MSC-derived exosomes, and immunomodulation [66]. Similarly, the emerging role of RPs in promoting angiogenesis, tumor growth, and metastasis is gaining recognition, although additional studies are required to fully elucidate their function in MM. The principal challenge moving forward will be the translation of these findings into routine clinical practice. Specifically, incorporating MSCs and RPs as prognostic tools could significantly improve patient monitoring and treatment strategies in MM. However, substantial obstacles remain, including the need to standardize evaluation methods for MSCs and RPs and to validate their prognostic relevance in larger, controlled studies. Furthermore, the feasibility of integrating these cellular markers into clinical workflows poses logistical and financial challenges that must be addressed. Despite these hurdles, the successful incorporation of MSCs and RPs into clinical practice could offer a more comprehensive approach to MM management, enabling more precise prognostication and tailored therapeutic interventions, ultimately improving patient outcomes.

## Figures and Tables

**Table 1:** Diagnosis of MM according to the criteria of the International Myeloma Working Group.

Criteria	Definition/Comment
Clonal bone marrow PCs ≥ 10%, or biopsy-proven bony or extramedullary plasmacytoma	The percentage of plasmatic cells (PCs) in the BM will ideally be calculated using a core biopsy specimen. Plasma cells’ clonality is established by demonstrating Kappa/Lambda light chain restriction by flow cytometry, immunohistochemistry, or immunofluorescence.
One or more defining events of Multiple Myeloma (MM)	Myeloma-defining events: Evidence of end-organ damage caused by the plasma cell proliferative disorder, specifically as follows:
	○ Hypercalcemia: serum calcium > 0.25 mmol/L (> 1 mg/dL) higher than the upper limit of normal or > 2.75 mmol/L (> 11 mg/dL). ○ Renal insufficiency: creatinine clearance < 40 mL/min or serum creatinine > 177 mol/L (> 2 mg/dL). ○ Anemia: hemoglobin value of > 2 g/dL below the lowest limit of normal, or a hemoglobin value < 10 g/dL. ○ Bone lesions: one or more osteolytic lesions on skeletal radiography, CT, or PET/CT.
	**Biomarkers of malignancy**
	○ 60% or greater clonal plasma cells on bone marrow examination. ○ Serum involved/uninvolved free light chain ratio of 100 or greater, provided the absolute level of the involved light chain is at least 100 mg/L. ○ More than one focal lesion on MRI that is at least 5 mm or greater in size.

**Table 2:** Genetic alterations associated with groups’ risk of MM.

Risk	Genetic Alteration	Gene/Chromosome Involved	Patients with Multiple Myeloma (MM) (%)
Standard Risk	Trisomies	Chromosomes 3, 5, 7, 9, 11, 15, and 19	42%
	t(11;14)	*CCND1*	14–15%
	t(6;14)	*CCND3*	5%
High Risk	t(4;14) *	*FGFR3* and *MMSET*	12–15%
	t(14;16)	*c-MAF*	3–4%
	t(14;20)	*MAFB*	<1.5%
	Trisomy 21	Chromosome 21	–
	Gain (1q21)	*CKS1B, BCL9, IL6R, MCL1, MUC1, PDZK1, ADAR. ILF2, among others* [15].	17–33%
	del(17p) **	*TP53*	6.6–11%

* The poor prognosis conferred by t(4;14) appears to be overridden by the presence of trisomies 3 and 5. ** The presence of trisomies with any IgH translocation improves the poor prognosis conferred by the del(17p) [13,14]. MM: multiple myeloma

## Data Availability

The data presented in this study are available on request from the corresponding author.
